# Supplementary material for: Content-rich biological network constructed by mining PubMed abstracts
Source: BMC Bioinformatics. 2004 Oct 8;5:147. doi: 10.1186/1471-2105-5-147 (PMC528731; doi:10.1186/1471-2105-5-147)
Supplement: Additional File 2 — The original results of the above study (non-essential files are deleted to keep the file size under the limit set by BMC bioinformatics). [file 1471-2105-5-147-S2.bz2 › chilibotAdditionalFile2/dip05/350ID8649415E4594/html/IL3_IL3RA.html]

 


 **IL3** and **IL3RA** 
  
Found 22 abstracts in PubMed, retrieved 05.  
 

 What does Google say? 
 PDF only 
| .edu only 

---

**Interactive relationship** (e.g. stimulation, inhibition, etc)

**Neutral relationship**- Freshly isolated CD123  [ **IL3RA** ]  pre DC showed typical plasmacytoid morphology and when cultured with IL 3  [ **IL3** ]  and CD40L for 72 hr, developed mature DC morphology.  Ref: 12829599 Blood< MedlineTA>Blood,

**Non-interactive relationship** (e.g. studied together, co-existance, homology, etc.)

- The interleukin 3  [ **IL3** ]  IL 3  [ **IL3** ]  receptor CD123  [ **IL3RA** ]  was used to identify basophils in the leukocyte mixture.  Ref: 12587990 Homeopathy, 2003
- Although blast cells lacked basophil specific antigens such as CDw17, CD88, and FcepsilonRI, an expression profile of cytokine receptors including CD116 GM CSF receptor, CD117 c kit, and CD123  [ **IL3RA** ]  IL 3  [ **IL3** ]  receptor alpha helped to define the cellular lineage in our case.  Ref: 11999362 Int J Hematol, 2002
- MATERIALS AND METHODS Based on data from 34 patients with hematologic malignancies, doses of CD34 cells and CD34 cell subsets, defined by the expression of HLA DR, CD38, CD117 c kit R, CD123  [ **IL3RA** ]  alpha subunit of IL 3  [ **IL3** ]  R, CD133 AC133, and CD90 Thy 1 antigens, were correlated with the number of short term i.e.  Ref: 11750108 Exp Hematol, 2001
